# Primary management of burn injuries: Balancing best practice with pragmatism

**DOI:** 10.4102/safp.v62i1.5202

**Published:** 2020-09-04

**Authors:** Nikki L. Allorto

**Affiliations:** 1 Department of Surgery, Faculty of Health, University of KwaZulu-Natal, Durban, South Africa; 2 Pietermaritzburg Burn Service, Greys Hospital, Pietermaritzburg, South Africa

**Keywords:** management of burns, blister controversy, first aid for burns, acute washing of the burn wound, fluid resuscitation, early enteral feeding, dressing the burn wound, analgesia

## Abstract

Management of burns is an often-neglected area in training from undergraduate to specialist level. There is, however, a high burden of injury that affects a largely vulnerable population, for example, children, the elderly and epileptics. This CPD article highlights that first aid should include cooling the burn with cool running tap water up to 3-hours post injury (Burnshield may be used if cool running water is not available); removal of all blisters facilitates accurate assessment of the burn size and depth; formulas exist for the resuscitation of acute burn injuries of more than 10% – 15% total body surface area and prophylactic antibiotics should not be administered to patients with acute burns as the prevention of infection should lie with good wound care (including good wound cleaning and the use of topical antimicrobial dressings). A standardised approach to pain management with an incremental pharmacological approach should be followed whilst considering other issues such as neuropathic pain, anxiety and depression.

## Introduction

Adhering to simple principles can have a significant impact on improving outcomes. These principles include: first aid for burns, the blister controversy and acute washing of the burn wound, fluid resuscitation and early enteral feeding, dressing the burn wound and analgesia.

## First aid for burns, the blister controversy and acute washing of the burn wound

Cooling the burn with cool running tap water has been shown to decrease cellular damage and oedema, reduce the inflammatory reaction with increased healing and decreased need for skin grafting. Although the ideal temperature of water is unknown, the duration shows maximum benefit when done for 20 min and is useful when done up to 3 h post burn.^1^ Cooling the burn with ice is detrimental and can lead to prolonged vasoconstriction. Other first aid components include immediate removal of clothing and jewellery, but any clothing melted or firmly adherent to the wound should be left to experienced personnel. Cover the burn wound with cling film or a clean non-adhesive dressing.^2^ Do not apply any other substance to the burn wound.^1^ Burnshield^®^ has previously been recommended by the South African Burn Society burn stabilisation protocol (2009)^1^ but more recent research has shown this to be inferior to cool running water (2017)^3^ as a first aid measure. However, where there is no running water available, it is not unreasonable to use Burnshield^®^. It must be clear that this is first aid and not a definitive care. The Burnshield^®^ should be removed on presentation to hospital in order for the burn to be assessed adequately. This should not be left on as a dressing until the next day.

**FIGURE 1:**
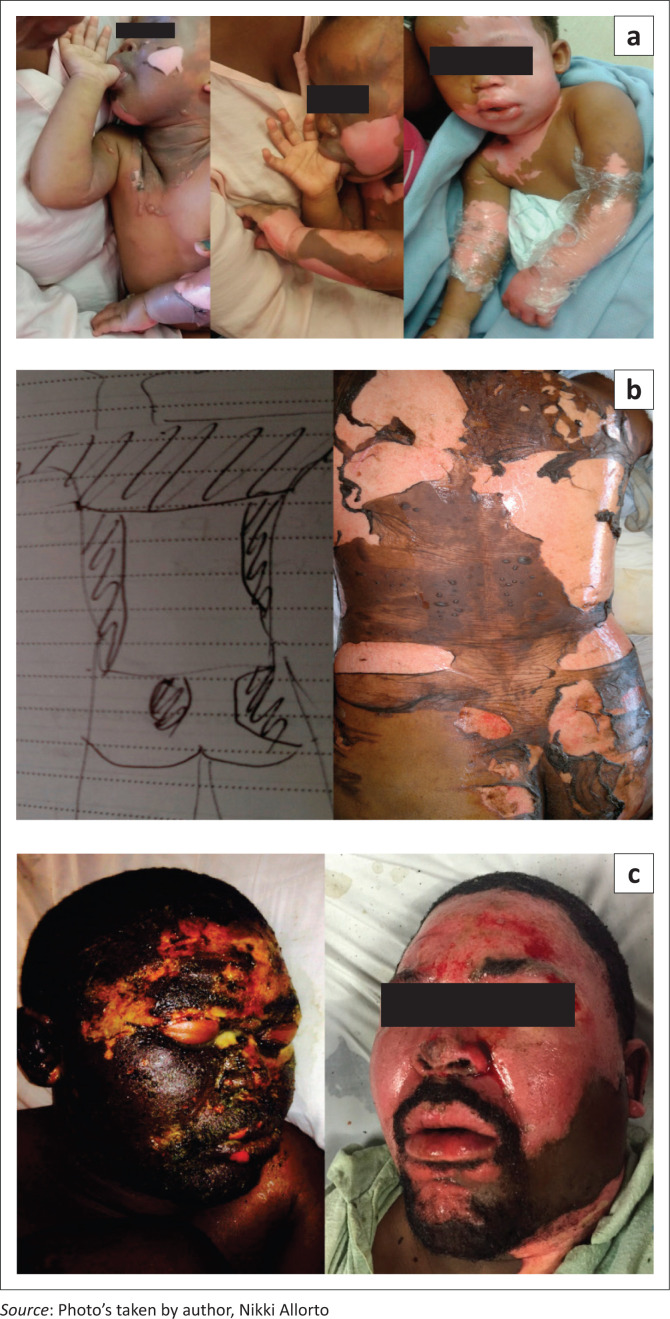
The importance of cleaning the burn wound: (a) the actual burn size becomes apparent only after removal of blisters and adequate cleaning, (b) perceived total body surface area by care provider not an accurate reflection of the burn as blisters and non-viable epithelium are not cleaned and (c) only once the wound has been cleaned properly, the superficial partial nature of the burn is appreciated.

The definitive management of any pathology depends on the diagnosis, which in the case of burns depends on the depth and total body surface area (TBSA) involved. This diagnosis cannot be attained if the wound is not adequately cleaned. Although the debate on deroofing or aspiration of blisters has raged on, a randomised controlled trial in 2017 demonstrated that neither showed superior outcome in healing rates, pain or scars.^4^ It is pertinent to note that the average size of burn surface area treated in this study was 3.5%. It is questionable, if damaged epithelium is left intact, how the diagnosis of depth and TBSA is made as demonstrated in Figure 1. This is particularly relevant in the case of the inexperienced care provider and the larger burn injuries. If the burn is larger than a thumbnail size, it is our protocol that the burn be cleaned of all blisters and debris. However, there is no evidence for what agent should be used in cleaning of the wound.^5^ The International Society for Burn Injuries recommends gentle washing as the most important component of burn wound cleansing^6^ rather than using an agent. Unpublished data from the Red Cross War Memorial Children’s Hospital has informed our local protocol of washing all acute burns on presentation with chlorhexidine gluconate warm soapy water to remove debris and blisters with the intention of an accurate burn diagnosis and the reduction in incidence of toxic shock syndrome, although this is anecdotal.

## Fluid resuscitation and early enteral feeding

There are a number of formulas to guide resuscitation, which belies that there is no perfect formula. It is important to remember the pathophysiology of burn injury in order to understand why the resuscitation occurs over 24 h. The burn injury mounts an inflammatory response leading to vasodilation and capillary leak into the extravascular space (hence the typical swelling) as well as losses through the open wound. These losses are ongoing and fluid resuscitation needs to be ongoing. There is no value in bolus dosing of fluid in the face of a normal blood pressure and also no need to rapidly correct the blood gas, which predictably shows a metabolic acidosis, the magnitude of which will be proportional to the size of the burn. The blood gas is not needed to guide the acute resuscitation and is labour-intensive in most low resource settings. Urine output is much simpler and more practical. It is therefore imperative that if the patient is to be fluid resuscitated, a urinary catheter must be inserted for monitoring. The Parkland formula is the most commonly used formula at 4 mL/kg/% but the modified Brooke formula at 2 mL/kg/%^7^ is our preferred protocol as doctors are comfortable giving more fluid for low urine output but reluctant to reduce the rate even if urine output is more than adequate.^8^ The complications of over resuscitation, for example, burn depth conversion are also under appreciated. The fluid used should be Ringers Lactate or equivalent such as Balsol or Plasmolyte and not Normal Saline because of the high chloride load. Titrate the fluid rate to achieve a urine output of not less than half a mL/kg/h and not more than 1 mL/kg/h. More than 1 mL/kg/h necessitates a decrease in the fluid rate and less than half a mL/kg/h necessitates an increase in the rate and not a bolus because the ongoing losses need to be addressed. Dextrose containing fluid should be given at maintenance rates for all children until they feed adequately. Fluid resuscitation is typically done for burns greater than 10% – 15% TBSA. In burns greater than 15% – 20% TBSA, this should be accompanied by insertion of a nasogastric tube for early enteral feeding. Nutritional requirements in larger burns are high and often exceed the patient’s appetite and ability to eat enough. Enteral feeding therefore guarantees the demand is met. Evidence also shows early feeding ameliorates the hypercatabolic response so typical of major burn injuries.^9^ Frequent questions arise around what to do with fluid at the end of the classically described resuscitation period at 24 h. If the patient is overloaded or euvolaemic, further intravenous fluid should not be given and the enteral feeds will suffice. Continue fluids after 24 h only if the patient is clinically dehydrated.

## Dressing the burn wound

Prophylactic antibiotics should not be administered to patients with acute burn injuries in an attempt to reduce the burn wound infection rates.^6^ The selective pressure leading to the development of resistant organisms will outweigh any potential short-term benefits. The prevention of infection should lie with good wound care that includes good wound cleaning (mechanical benefit of removing the bioburden) and use of topical antimicrobial dressings if available. Whilst silver sulphadiazine has long been associated with burn wound care, it is shown to be associated with poorer healing outcomes compared with other options.^10^ There are many modern options available with none shown to be superior to another. Rather it is a matter of practicality. Even something as simple as paraffin gauze if nothing else is available will be reasonable provided some basic principles are adhered to. These principles are anecdotal as good evidence in this field is lacking.

The primary layer in all wounds must lie flush with the wound bed. The secondary layer and fixation should contribute to the primary layer being flush with the wound and to hold the dressing in place for the prescribed duration. This is particularly pertinent where burns are located in awkward areas such as the axilla, neck and groin for example. Use of Hypafix^®^, for example, is extremely useful as it is sticky but breathable. Occlusive dressings often promote bacterial proliferation and exacerbate local infection and should not be used to secure dressings. For hand burns, fingers should be individually dressed. This prevents the development of syndactyly as well as promotion of using the hands in activities of daily living and allows the occupational therapist to perform both passive and active mobilisation. Take care to cut each layer to the appropriate size. Avoid doing bulky dressings. The old silver sulphadiazine ‘glove’ although had good intentions is problematic. The normal skin becomes macerated and the hand tends towards the position of the burn claw hand with flexion at the interphalangeal joints and dorsiflexion at the metacarpophalangeal joints because of skin and tendon shortening. This is not functional and results in loss of grasp function. Use of the intrinsic plus splint is more useful, which maintains flexion to 90 degrees at the metacarpophalangeal joints and extension of the fingers. Preservation of the first web space is also important and assists in maintaining the grasp function. The perineum is another challenging area and the wounds are often soiled by stool. Minor wounds will heal anyway. Larger wounds may require medically induced constipation whilst healing. In children, antiseptic creams or pastes lining the nappy and changed with each nappy change is a practical solution.

## Analgesia

Burn injuries are painful. Leaving a patient in pain has physiological and psychological consequences and therefore good pain management is one of the tenets of burn care.^11^ There are various components of pain including acute pain that is background or breakthrough, and pain that is related to procedures such as dressing change. Chronic pain is a reality for a significant number of patients and other components such as pruritis, anxiety, depression and acute or post-traumatic stress disorder also need consideration.

Ketamine is a great procedural analgesic for dressing change and has been described in a number of settings.^11^ It is extremely versatile and can be administered orally, intramuscularly and intravenously. A common challenge is tachyphylaxis, which means a diminishing response to successive doses. This tachyphylaxis is an acute response rather than a slow progressive response, which is a tolerance typically seen with opioids. Therefore, procedural analgesia should be given under supervision and repeated doses be given as necessitated by pain scores at the time of the dressing. A standard dose of ketamine for the duration of the patient’s care is not appropriate and escalating doses are normal. Ketamine is ideal for many settings including use in the ward or the outpatient department as only monitoring of heart rate and saturation is required. More recently, methoxyflurane (Penthrop^®^) is available in the country. This is an inhalational analgesic with rapid onset of action for short-term analgesia. It belongs to the fluorinated hydrocarbon group of anaesthetic agents. The analgesic potency is high in low concentrations compared with other volatile anaesthetic agents. Pain relief begins after 6–8 breaths and continues for several minutes after inhalation has ceased.^12^ Methoxyflurane is supplied as a 3 mL vial, which is poured onto the wick of a hand-held inhaler. Once the wick is saturated with methoxyflurane, the patient then inhales the methoxyflurane via the mouth piece. It can be administered without monitoring^11^ and is a good option for procedural analgesia in selected patients. Small children and anxious patients are not good candidates for this. Ketamine and methoxyflurane are good options for the non-anaesthetist doing burn dressing changes in a variety of low care or outpatient settings and should be widely utilised. Simple analgesics such as paracetamol and even opioids are insufficient for procedural analgesia.

Background analgesia is the other component to be addressed. It is dynamic and may diminish as healing takes place and may worsen postoperatively, for example, if the wound is complicated by infection. Prescription should, therefore, be a dynamic process and be changed with the patients’ changing needs. A standardised approach to pain management with an incremental pharmacological approach should be followed.^13^ A locally developed practical guide for a stepwise approach to background pain as well as procedural ketamine use can be found at www.burncarethepmbway.com, with the pain protocol having undergone a modified Delphi process and currently under review for publication.

Adjuvant therapies such as clonidine and gabapentin are extremely beneficial if available and useful where pruritus is debilitating and antihistamines alone are inadequate to achieve relief of symptoms. When pain is difficult to control or seems refractory, consider other problems, for example, anxiety and depression. The use of long acting benzodiazepines and or antidepressants may be needed. Use of adjuvant therapies such as psychosocial support, aromatherapy and music therapy can be beneficial but is often limited by accessibility.
